# Correlates of Performance of Healthcare Workers in Emergency, Triage, Assessment and Treatment plus Admission Care (ETAT+) Course in Rwanda: Context Matters

**DOI:** 10.1371/journal.pone.0152882

**Published:** 2016-03-31

**Authors:** Celestin Hategekimana, Jeannie Shoveller, Lisine Tuyisenge, Cynthia Kenyon, David F. Cechetto, Larry D. Lynd

**Affiliations:** 1 School of Population and Public Health, Faculty of Medicine, University of British Columbia, Vancouver, BC, Canada; 2 Collaboration for Outcomes Research and Evaluation, Faculty of Pharmaceutical Sciences, University of British Columbia, Vancouver, BC, Canada; 3 Department of Pediatrics, University Teaching Hospital of Kigali, Kigali, Rwanda; 4 Division of Neonatal-Perinatal Medicine, Children's Hospital at London Health Sciences Centre, London, ON, Canada; 5 Schulich School of Medicine and Dentistry, Department of Anatomy & Cell Biology, Western University, London, ON, Canada; 6 Center for Health Evaluation and Outcome Sciences, Providence Health Research Institute, Vancouver, BC, Canada; Johns Hopkins Bloomberg School of Public Health, UNITED STATES

## Abstract

**Background:**

The Emergency, Triage, Assessment and Treatment plus Admission care (ETAT+) course, a comprehensive advanced pediatric life support course, was introduced in Rwanda in 2010 to facilitate the achievement of the fourth Millennium Development Goal. The impact of the course on improving healthcare workers (HCWs) knowledge and practical skills related to providing emergency care to severely ill newborns and children in Rwanda has not been studied.

**Objective:**

To evaluate the impact of the ETAT+ course on HCWs knowledge and practical skills, and to identify factors associated with greater improvement in knowledge and skills.

**Methods:**

We used a one group, pre-post test study using data collected during ETAT+ course implementation from 2010 to 2013. The paired t-test was used to assess the effect of ETAT+ course on knowledge improvement in participating HCWs. Mixed effects linear and logistic regression models were fitted to explore factors associated with HCWs performance in ETAT+ course knowledge and practical skills assessments, while accounting for clustering of HCWs in hospitals.

**Results:**

374 HCWs were included in the analysis. On average, knowledge scores improved by 22.8/100 (95% confidence interval (CI) 20.5, 25.1). In adjusted models, bilingual (French & English) participants had a greater improvement in knowledge 7.3 (95% CI 4.3, 10.2) and higher odds of passing the practical skills assessment (adjusted odds ratio (aOR) = 2.60; 95% CI 1.25, 5.40) than those who were solely proficient in French. Participants who attended a course outside of their health facility had higher odds of passing the skills assessment (aOR = 2.11; 95% CI 1.01, 4.44) than those who attended one within their health facility.

**Conclusions:**

The current study shows a positive impact of ETAT+ course on improving participants’ knowledge and skills related to managing emergency pediatric and neonatal care conditions. The findings regarding key factors influencing ETAT+ course outcomes demonstrate the importance of considering key contextual factors (e.g., language barriers) that might affect HCWs performance in this type of continuous medical education.

## Introduction

Rwanda has a population of more than 11 million people, of which 48% are younger than 18 years old [1]. There are approximately 410,100 births per year and only about 20 pediatricians were working in the country as of 2011, mostly in national referral hospitals [2–3]. The healthcare workforce in district hospitals–the backbone of the health care system–is primarily comprised of generalist physicians and secondary school trained nurses (A2-level, the lowest level of nursing training available) [4]. These physicians and nurses are often required to handle complicated pediatric and neonatal emergencies in the absence of specialists. Healthcare workers (HCWs) have expressed concerns about gaps in their training and their lack of confidence with regards to dealing with maternal newborn and child emergency conditions [5].

District hospitals are the first-line referral sites in low and middle resource countries, and thus it is critical that they be capable of providing effective, efficient and high quality care to all patients [6, 7]. Therefore, additional training is needed to better prepare HCWs in these facilities to provide effective emergency pediatric and neonatal care. While the implementation of advanced pediatric life support courses has been advocated for in these countries to facilitate the achievement of the fourth Millennium Development Goal (i.e. MDG 4: reducing under-five mortality by two-thirds between 1990 and 2015), many existing pediatric advanced life support management guidelines in limited-resource settings are not standardized. Furthermore, systematic approaches to patient assessment and categorization of disease is rare, and current pediatric advanced life support training courses tend to be designed and implemented without sufficient attention to the local context and its associated resource constraints [8].

In an effort to ameliorate these deficiencies, the Emergency, Triage, Assessment and Treatment plus Admission care (ETAT+) course was developed in East Africa for HCWs caring for acutely ill newborns and children. The ETAT+ course is a locally adapted pediatric advanced life support course that offers intensive, competency-driven training over a five-day period that aims to improve pediatric emergency and admission care in the initial 24 hours of hospitalization [9]. The course covers the recognition and initial management of the most common medical causes of pediatric hospital admission in East Africa (Table 1). It was introduced in Rwanda in 2010 to contribute to the reduction of the mortality rate in children less than five years [10].

**Table 1:** ETAT+ course content.

Triage
Infant and child resuscitation
Recognition of a sick child
Diarrhea/dehydration and shock
Newborn care–preterm, jaundice, feeding, sepsis
Pneumonia
Malaria
Asthma
Severe malnutrition
Meningitis
Hypoglycemia
Convulsions
Prescribing and procedures–oxygen, lumbar puncture, intra-osseous infusion

ETAT+, Emergency Triage Assessment and Treatment plus Admission care

Clinical practice guidelines, including ETAT+, are developed to help HCWs deliver the best care to patients by combining the best available evidence on the management of diseases into specific recommendations for care. Nevertheless, evidence-based guidelines are rarely implemented with perfect fidelity under real-world conditions. Therefore, evaluations of the real-world experiences associated with implementing such guidelines are important in terms of identifying potential barriers to successful implementation as well as identifying factors that contribute to their successful adoption and scale-up over time.

With these aims in mind, we analyzed data gathered during the implementation of the ETAT+ course for HCWs in Rwanda to evaluate the performance of HCWs in knowledge acquisition and skills assessments with a goal of gathering evidence that would be used to inform the course tailoring and revisions (e.g., course content; scope and sequencing of training modules; program delivery and teaching methods). The objectives of this study were therefore to: (i) determine the effect of ETAT+ course on improvement in knowledge and skills related to pediatric advanced life support management among HCWs in Rwanda; (ii) identify the components of the ETAT+ course in which HCWs demonstrated the largest and most persistent gaps in knowledge or skills; and (iii) identify key factors associated with HCWs performance on the ETAT+ course knowledge and skills assessments.

## Materials and Methods

### Overview of ETAT+ course implementation

The ETAT+ course consists of lectures, demonstration and practical sessions and case scenarios using manikins, a hospital audit including a review of selected patient medical records within the specific institution where the course is occurring, and assessment and feedback. The lectures are short and topic specific, and are accompanied by demonstrations [9]. Discussions and hands on practice take place in small groups of 5–7 participants where they work in inter-professional groups with nurses, midwives and physicians all learning together. Course preparation materials are provided to participants prior to the start of the course. These materials include an invitation letter highlighting course venue and expectation, timetable, *Rwandan basic pediatric protocols* [11], *WHO pocket book of hospital care for children* [12], and a pre-course knowledge assessment. Attendance for the entire five days is compulsory to receive the certification.

**Fig 1:**
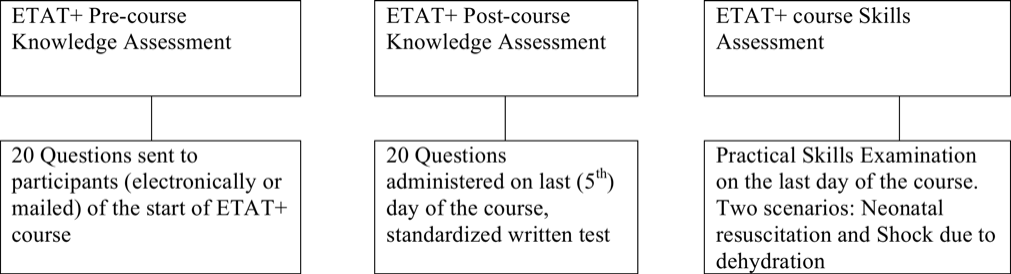
ETAT+ Participant Assessment Schedule.

For the pre-course knowledge assessment, all participants are required to complete a standard knowledge assessment of 20 multiple-choice questions (MCQs) before the start of the course with access to the course materials, while post-course knowledge assessment are completed under closed-book exam conditions (Fig 1). The pre-course and post-course MCQs are different but test the same concept. The assessments are taken in English or French depending on language proficiency and preference of participants. The knowledge assessment score is the proportion of correct answers. Upon completion of the course, all participants are required to retake the knowledge assessment. Moreover, the participants are assessed on two of the clinical skills scenarios that are discussed during the course; however, they have no prior knowledge of the clinical skills selected for assessment. Information pertaining to participant characteristics is also collected at the start of the course. ETAT+ course instructors train and assess participants and the senior instructor monitor instructors’ performance during training and assessment.

Participants are also assessed according to standardized criteria on two clinical skills scenarios—one pertaining to neonatal resuscitation and the other focusing on the management of a severely ill child with shock from dehydration. The practical skills assessment is done using an Objective Structured Clinical Examination (OSCE) format with a clinical scenario and a standardized checklist. To pass the skills assessment, participants are required to pass both scenarios. If they fail both scenarios on their first attempt, they are judged to fail and are not allowed to redo the scenarios. However, if participants do not meet all standardized criteria in just one scenario, they are allowed to retake the same clinical skills scenario after receiving feedback. If all standardized criteria are met after the retake, they are deemed to pass the skills assessment; otherwise, they fail the assessment.

The implementation of the ETAT+ course in Rwanda is overseen by the Rwanda Pediatric Association, which also trains ETAT+ course instructors.

### Study Design

The current analysis was a quantitative assessment of the improvement in emergency pediatric and neonatal care knowledge and skills immediately after an intensive full five-day of ETAT+ course–held between 2010 and 2013 –for HCWs in Rwanda using one group pre- post-test, and post-test only study designs. The pre-post test design was used to assess the impact of ETAT+ on change in knowledge (i.e. difference between pretest score and post test score), while the post-test only design was used to assess the impact of the course on skills performance as there was no skills assessment conducted prior to the course. Program implementers assumed that pre-test skills assessments would have been futile, as all participants would likely perform very poorly since they were not familiar with standardized criteria required to pass the assessment.

### Analytic Sample

As of part of a national strategy to reduce under-five mortality, ETAT+ course has been implemented in many health facilities in Rwanda. This study includes all district hospitals and national referral hospitals in which ETAT+ course was implemented between 2010 and 2013. As of 2010, Rwanda had 40 district hospitals and 4 national referral hospitals (http://moh.gov.rw/fileadmin/templates/HMIS_Docs/MOH_Annual_booklet__2010.pdf). The hospitals were purposefully selected to ensure reasonable regional representation while considering availability of funds. The ETAT+ course participants were also purposefully selected to ensure those working in newborn and child health related departments (e.g. pediatrics, maternity, emergency room) were trained as they were expected to use the knowledge and skills gained when they return to their affiliated hospitals to improve emergency care provided to sick newborns and children.

The current analysis includes HCWs (i.e. nurses, midwives, generalist and specialist physicians) who were working in health facilities in Rwanda and enrolled in an ETAT+ course between 2010 and 2013, and who completed the pre- and post-course knowledge assessments as well as ETAT+ practical skills assessment. This analysis excludes participants who did not complete the full five-day course, final year medical students, and HCWs who were working abroad (i.e. outside Rwanda) but completed the course while in Rwanda.

### Study Variables

The outcomes for this study included pre-course knowledge score, post-course knowledge score, change in knowledge, and practical skills score. Pre-course and post-course knowledge assessment scores were defined as the percentage of correct answers in the pre- and post-course knowledge assessments, respectively. Change in knowledge outcome was defined as the difference between pre-course and post-course knowledge scores, while practical skill-based competency was defined as passing both the neonatal resuscitation and shock scenarios. ETAT+ certification requires achieving a score of at least 50% on post-course knowledge assessment and passing the practical skills assessment. These standards reflect the criteria that are required for certification in ETAT+ course [13].

Variables (see Table 2) included in the current analysis were hypothesized to be associated with improvement in knowledge or scores on the practical skills assessment were based on existing literature (e.g., profession, years since graduation/experience) [14] or were identified during the course implementation as potentially important factors (e.g., course location, type of materials, timing of delivery of materials, language proficiency).

**Table 2:** Characteristics of Study Sample.

		Overall Study Sample	
		n = 374	%
***Variables hypothetisized to be associated with performance in the ETAT+ course***			
***Health Facility***			
*Type of facility*	Referral hospital	44	11.8
	District hospital	330	88.2
*Location*	Rural	120	32.1
	Urban	254	67.9
***Healthcare provider***			
*Profession*	Nurse	245	65.5
	Midwife	34	9.1
	Generalist MD	68	18.2
	Specialist MD	27	7.2
*Sex*	Female	234	62.5
	Male	140	37.5
*Language proficiency*	French	199	53.2
	English	30	8.0
	Bilingual (French and English)	145	38.8
*Years since graduation*	Less than or equal to one year	40	10.7
	More than one year	334	89.3
*Department of affiliation*	Pediatric department	201	53.7
	Maternity department	83	22.2
	Emergency room	47	12.6
	Other departments*	43	11.5
*Pre-course knowledge score*	Percentage of correct answers		
*Previous attendance of ETAT+ course*	Yes	12	3.2
	No	362	96.8
***ETAT+ course organization***			
*Location*	Within health facility of participants affiliation (on-site),	48	12.8
	Outside of health facility of participants affiliation (off-site)	326	87.2
*Formant of course materials received*	Hard copy	207	55.4
	e-copy	167	44.6
*Time the course materials were received*	Received one week before the course began	166	44.4
	3–4 weeks before the course began	208	55.6

* Internal Medicine, Surgery, Pharmacy, and Administration

ETAT+, Emergency Triage Assessment and Treatment plus Admission care

### Statistical Analysis

Descriptive statistics including frequency (and percentages) for categorical variables and mean (and standard deviation, SD) for continuous variables were computed and reported. Change in knowledge scores was assessed by comparing pre- and post-course scores using a paired t-test. We used mixed effects linear and logistic regression models to assess the correlates of knowledge improvement and practical skills performance among HCWs who participated in the ETAT+ course, while accounting for clustering of HCWs in hospitals. We performed bivariate analyses to investigate the relationship between each explanatory variable and outcomes (i.e., change in knowledge scores and skills performance) to see which variables pass an initial screening at α set to p = 0.2 as model entry significance level. All potential predictors independently associated with outcomes (at an α level of 0.2) during bivariate analysis were retained for further exploration in the multivariate regression models. Backward selection was then applied (p>0.05) to select the most parsimonious models. We achieved model selection by minimizing Akaike Information Criterion (AIC) and Bayesian Information Criterion (BIC) while keeping p values for covariates below 0.05. For logistic models, associations are reported as odds ratios (OR) and 95% confidence interval (CI) while regression coefficient (ß) and 95% CI are reported for the linear models. All statistical analyses were performed with RStudio version 3.2.2 (RStudio Inc., Boston, MA, USA).

### Ethics Statement

This study received approval from the Research Ethics Board of the University of British Columbia and from the Rwanda Pediatric Association. The current study used existing data that were collected during the implementation of the ETAT+ course in Rwanda. As such the consent was not obtained and participants’ information were anonymized and de-identified prior to analysis.

## Results

### Characteristics of Study Sample

Of 615 individuals who were trained in the ETAT+ course between 2010 and 2013, 241 (39.2%) participants were excluded from the current analysis: 1.8% (n = 11) were HCWs who did not complete the full five-day course; 36.0% (n = 222) were final year medical students but were not yet in practice; and 1.3% (n = 8) were HCWs who were working abroad (i.e. outside Rwanda) but completed the course while in Rwanda. Therefore, the final analytic sample included 374 HCWs from 24 health facilities including three of the four national referral hospitals and 21 of Rwanda’s 40 district hospitals.

The overall study sample was unevenly distributed across profession, with more nurses than doctors (generalists and specialists) (Table 2). Most course participants were from district hospitals, were more than one year post graduation, or attended an ETAT+ course held outside their home healthcare facility. Only 3.2% of participants reported having previously attended an ETAT+ course. Two-thirds of the participants were female and 67.9% were working in an urban hospital, while 53.7% of the participants were working in a pediatric department when they attended the course and 53.2% identified as fluent in French (Table 2).

### Knowledge Assessment Performance

**Fig 2:**
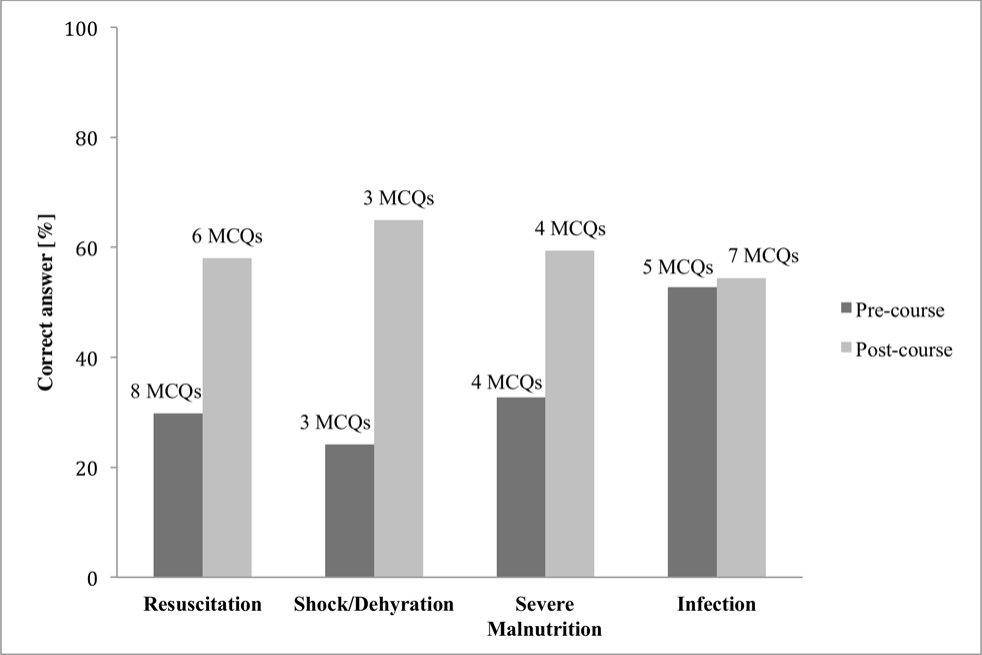
Knowledge improvement per ETAT+ course components assessed in the knowledge assessments. MCQs, multiple choice questions.

Only 77 (20.6%) participants achieved a score of 50% or more on the pre-course knowledge assessment, versus 292 (78.0%) on the post-course knowledge assessment. The overall mean (SD) score on the pre- and post-course knowledge assessment was 35.3 (19.7) and 58.0 (13.4), respectively, resulting in an overall mean change in knowledge assessment scores of 22.8 (20.4–25.1) (p<0.0001). Nearly all components of the knowledge assessment appear to contribute equally to failure of both pre- and post-test (Fig 2). The participants demonstrated greater knowledge gaps in resuscitation and management of shock/dehydration and severe malnutrition compared to the management of infection, and thus the improvement in these three components was greatest, with some improvement demonstrated in the infection management component (Fig 2).

Performance in pre-course knowledge assessment varied, with midwives, those who had previously attended ETAT+ course, those who identified as proficient in English, and those who received the course materials more than a week before the start of the course achieving a higher score, on average (Table 3). Likewise, performance on post-course knowledge assessment differed with higher average scores achieved by specialist MDs, those who completed their professional training more recently, those who had attended an ETAT+ course previously, and those who identified as proficient in both French and English (Table 3). Greater overall improvement in knowledge scores was evident among specialist doctors, those who were working in national referral hospitals or in other departments when they attended the course, those who had not attended the course before, those who reported being proficiently bilingual, those who received the course materials one week of the start of the course, and those who attended the course outside of their health facility (Table 3).

**Table 3:** Performance on Knowledge and Practical Skills Assessments.

	Knowledge Assessment					Skills Assessment	ETAT+ Certification
	Pre-course		Post-course		Knowledge Change		
	Mean score (SD)	Pass rate^¶^ %	Mean score (SD)	Pass rate^¶^ %	Mean score (95% CI)	Pass rate ^¥^ %	Achieved^†^ %
**Profession**							
Nurse	32.4 (19.9)	16.3	55.1 (13.0)	71.4	22.9 (20.1–25.8)	71.5	59.6
Midwife	46.0 (23.1)	41.1	55.1 (8.9)	88.2	11.9 (3.5–20.3)	94.2	88.2
Generalist MD	38.6 (18.8)	23.5	63.2 (12.5)	89.7	24.0 (18.2–29.8)	91.2	83.8
Specialist MD	40.1 (16.8)	25.9	72.0 (10.9)	96.3	31.9 (25.2–38.6)	96.3	96.3
**Sex**							
Female	34.9 (20.3)	20.5	56.9 (12.9)	77.7	22.0 (19.0–25.0)	78.2	69.7
Male	36.2 (20.0)	20.7	59.7 (14.0)	78.5	24.1 (20.3–27.9)	80.0	68.6
**Health facility**							
District hospital	35.6 (21.0)	20.9	57.2 (12.9)	76.6	21.9 (19.3–24.5)	77.9	67.2
National referral hospital	33.9 (13.2)	18.2	63.3 (15.3)	88.6	29.4 (24.9–34.0)	86.4	84.1
**Hospital location**							
Rural	34.0 (20.6)	18.3	57.4 (13.0)	75.8	23.3 (18.9–27.7)	76.7	63.3
Urban	35.8 (19.2)	21.6	58.3 (13.6)	79.1	22.5 (19.7–25.3)	79.9	72.0
**Department**							
Emergency Room	36.0 (20.8)	23.4	54.8 (12.7)	70.2	19.0 (12.2–25.8)	80.9	61.7
Maternity	36.3 (21.9)	24.1	56.3 (12.0)	77.1	20.6 (15.3–25.9)	78.3	68.7
Pediatrics	35.6 (19.5)	19.4	59.4 (13.9)	80.5	23.8 (20.6–27.0)	80.6	72.3
Other	32.2 (19.5)	16.2	58.3 (14.0)	76.7	26.1 (20.2–32.0)	69.8	62.8
**Years from graduation**							
< = 1 year	40.9 (25.0)	32.5	65.4 (9.9)	100	24.8 (15.9–33.6)	97.5	97.5
> 1 year	34.7 (19.5)	19.2	57.0 (13.5)	75.4	22.5 (20.1–24.9)	76.7	65.8
**Previously attended ETAT+**							
No	34.8 (20.0)	19.8	57.6 (13.4)	77.3	23.0 (20.6–25.3)	78.1	68.2
Yes	50.0 (22.0)	41.6	66.6 (10.5)	100	16.6 (0.5–32.8)	100	100.0
**Language**							
French	32.2 (19.9)	14.5	54.0 (13.1)	67.3	22.2 (18.9–25.5)	68.2	53.8
English	43.7 (21.2)	36.6	59.8 (14.6)	76.6	17.2 (7.7–26.7)	86.7	73.3
Bilingual	38.4 (19.7)	25.5	63.1 (11.8)	93.1	24.6 (21.0–28.2)	91.7	89.6
**Location of ETAT+ course**							
Within facility	39.3 (27.5)	33.3	54.0 (13.4)	64.5	14.6 (6.1–23.2)	62.5	50.0
Outside facility	34.6 (18.2)	18.7	58.6 (13.3)	80.0	23.9 (21.6–26.3)	81.3	72.1
**Format of course materials received**							
Hardcopy	36.2 (20.7)	20.2	59.9 (14.0)	74.0	20.8 (17.3–23.8)	95.8	65.2
e-copy	34.8 (19.9)	20.9	56.6 (12.9)	83.2	25.5 (22.1–28.9)	65.2	74.2
**Time receipt course materials**							
1 week before the course	28.7 (13.5)	7.8	59.5 (13.0)	79.5	30.7 (28.1–33.3)	81.3	71.0
3–4 weeks before the course	41.8 (23.2)	30.7	56.5 (13.7)	76.9	16.4 (12.9–19.9)	76.9	67.8

¶ Passing pre- and post-course knowledge assessments require achieving a score of at least 50% on pre- and post-course knowledge assessments, respectively.

¥ Passing practical skills assessment requires passing both the neonatal resuscitation and shock scenarios.

† ETAT+ certification requires achieving a score of at least 50% on post-course knowledge assessment and passing the practical skills assessment.

ETAT+: Emergency, Triage, Assessment and Treatment plus Admission care; SD, standard deviation; CI, confidence interval.

### Performance on Skills Assessment

The pass rates for neonatal resuscitation and shock management scenarios were 85.0% (n = 318) and 82.4% (n = 308), respectively, while the overall pass rate on the skills assessment (i.e. passing both neonatal resuscitation and shock scenarios) was 78.6% (n = 294). Performance on the skills assessment varied with all participants who had previously attended the course and almost all of those who were one year away from graduation passing the skills assessment (Table 3).

**Fig 3:**
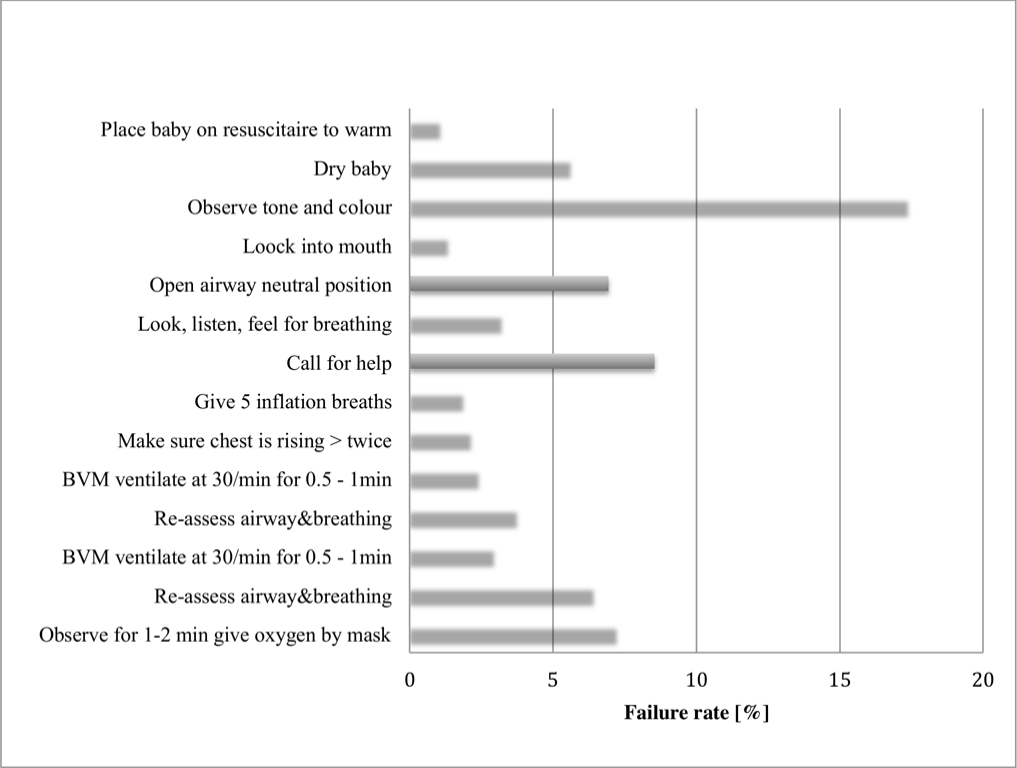
Rate of failure per each criterion assessed in the scenario on neonatal resuscitation. BVM, bag valve mask.

**Fig 4:**
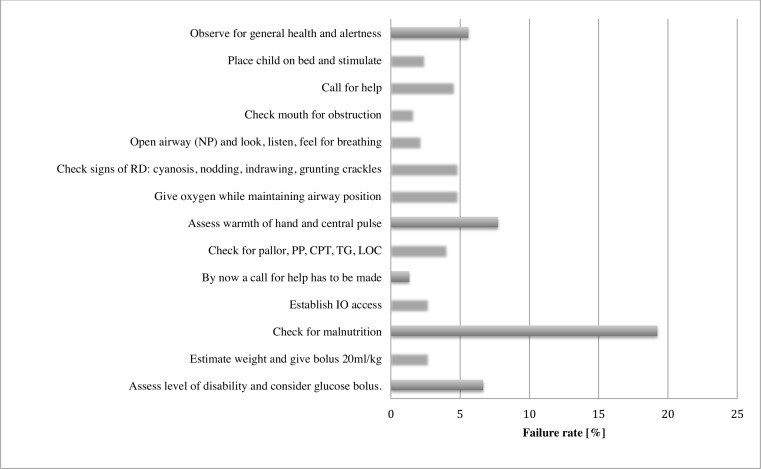
Rate of failure per each criterion assessed in the scenario on management of shock due to dehydration. CRT, capillary refill time; IO, intra-osseous; NP, neutral position; PP, peripheral pulse; TG, temperature gradient; LOC, level of consciousness; RD, respiratory distress.

With respect to reasons for failing the practical skills assessment, two components (observing muscle tone and color and calling for help) were most often missed in the neonatal scenario (Fig 3), while checking for malnutrition was the most frequent component missed in the shock scenario (Fig 4).

### Factors associated with HCWs’ Performance in the ETAT+ course Knowledge and Skills Assessments

Health facility related characteristics (i.e. type and location of health facility) were not associated with either improvement in knowledge (Table 4) or performance in practical skills assessment (Table 5).

**Table 4:** Crude and adjusted linear regression analysis of factors associated with improvement in knowledge assessment*.

	Pre-course knowledge score		Post-course knowledge score		Change in Knowledge	
	Crude ß coefficient (95% CIs)	Adjusted ß coefficient (95% CIs)	Crude ß coefficient (95% CIs)	Adjusted ß coefficient (95% CIs)	Crude ß coefficient (95% CIs)	Adjusted ß coefficient (95% CIs)
**Profession**						
Nurse	Reference		Reference	Reference	Reference	Reference
Midwife	5.9 (-0.1, 12.0)		1.5 (-2.9, 6.1)	-1.37 (-5.85, 3.05)	-4.1 (-11.6, 3.2)	-0.9 (-5.4, 3.5)
Generalist	5.0 (0.6, 9.4)		8.0 (4.7, 11.3)	6.58 (3.35, 9.79)	3.8 (-2.4, 8.4)	6.5 (3.2, 9.7)
Specialist	7.4 (0.2, 14.7)		17.5 (12.2, 22.7)	14.78 (9.49, 20.10)	9.6 (0.7, 18.5)	13.5 (8.3, 18.9)
**Sex**						
Female	Reference		Reference		Reference	
Male	2.0 (-1.5, 5.6)		3.6 (0.8, 6.4)		1.6 (-2.7, 5.9)	
**Health facility**						
District hospital	Reference		Reference		Reference	
National referral hospital	-0.4 (-13.4, 12.5)		5.7 (-0.2, 11.5)		3.9 (-10.7, 18.3)	
**Hospital location**						
Rural	Reference		Reference		Reference	
Urban	1.0 (-7.9, 9.7)		0.5 (-3.8, 4.8)		-1.1 (-11.0, 8.8)	
**Department**						
Emergency Room	Reference		Reference		Reference	
Maternity	-2.9 (-8.8, 3.0)		2.2 (-2.4, 7.0)		5.3 (-1.9, 12.5)	
Pediatrics	-2.1 (-7.6, 3.2)		2.8 (-1.4, 7.2)		4.2 (-2.3, 10.8)	
Other^¶^	-1.8 (-8.7, 5.0)		4.3 (-1.1, 9.8)		6.7 (-1.6, 15.1)	
**Years from graduation**						
< = 1 year	Reference		Reference	Reference	Reference	
> 1 year	-4.7 (-10.3, 0.8)		-8.9 (-13.2, -4.5)	-6.70 (-10.90, -2.51)	-4.4 (-11.3, 2.3)	
**Previously attended ETAT+**						
No	Reference	Reference	Reference		Reference	
Yes	17.8 (8.0, 27.5)	15.7 (5.9, 25.4)	10.0 (2.2, 17.7)		-7.2 (-19.2, 4.8)	
**Language**						
French	Reference	Reference	Reference	Reference	Reference	Reference
English	10.9 (4.4, 17.5)	10.8 (4.4, 17.3)	5.8 (0.8, 10.7)	4.24 (-0.44, 8.92)	-5.2 (-13.3, 2.8)	4.3 (- 0.5, 9.0)
Bilingual	6.3 (2.5, 10.1)	5.1 (1.3, 8.9)	9.7 (6.8, 12.5)	5.89 (2.84, 8.89)	3.6 (-1.0, 8.3)	7.3 (4.3, 10.2)
**Location of ETAT+ course**						
Within facility	Reference		Reference		Reference	
Outside facility	-7.1 (-23.6, 8.8)		4.9 (-1.6, 11.6)		12.1 (-4.9, 29.3)	
**Course Materials**						
Hardcopy	Reference		Reference		Reference	
e-copy	-7.4 (-15.5, 0.4)		1.9 (-2.1, 5.9)		6.7 (-1.9, 15.5)	
**Time of receipt of course materials**						
1 week	Reference	Reference	Reference		Reference	Reference
3–4 weeks	4.9. (0.2, 9.9)	5.8 (1.1, 10.6)	-1.8 (-5.2, 1.4)		-7.0 (-13.0, -1.2)	-2.1 (-5.2, -0.9)
**Pre-course assessment**						
Knowledge score			0.1 (-0.0, 0.1)		-0.9 (-1.0, -0.8)	-1.0 (-1.1, -0.9)

* Adjusted for clustering of healthcare workers in hospitals

^¶^ Internal Medicine, Surgery, Pharmacy, and Administration

**Table 5:** Crude and adjusted logistic regression exploring factors associated with skills performance*.

	Practical Skills Performance	
	Crude Odds Ratios (95% CIs)	Adjusted Odds Ratios (95% CIs)
**Profession**		
Nurse	Reference	Reference
Midwife	3.97 (1.15, 13.65)	3.12 (0.85, 11.37)
Generalist	4.33 (1.76, 10.62)	3.50 (1.40, 8.73)
Specialist	10.13 (1.32, 77.55)	4.87 (0.60, 39.32)
**Sex**		
Female	Reference	
Male	1.09 (0.64, 1.84)	
**Health facility**		
District hospital	Reference	
National referral hospital	1.70 (0.62, 4.64)	
**Hospital location**		
Rural	Reference	
Urban	1.05 (0.55, 2.01)	
**Department**		
Emergency Room	Reference	
Maternity	0.80 (0.32, 1.98)	
Pediatrics	0.94 (0.41, 2.16)	
Other^¶^	0.57 (0.21, 1.55)	
**Years from graduation**		
More than one year	Reference	Reference
Less than or equal to one year	14.13 (1.89, 105.56)	9.62 (1.20, 76.85)
**Previously attended ETAT+**		
No	Reference	
Yes	1.63 (0.00, 6.8)	
**Language**		
French	Reference	Reference
English	3.08 (1.03, 9.20)	2.31 (0.74, 7.23)
Bilingual	5.25 (2.71, 10.18)	2.60 (1.25, 5.40)
**Location of ETAT+ course**		
Within facility	Reference	Reference
Outside facility	2.55 (1.32, 4.91)	2.11 (1.01, 4.44)
**Course Materials**		
Hardcopy	Reference	
e-copy	1.21 (0.67, 2.20)	
**Time of receipt of course materials**		
3–4 week	Reference	
1 week	1.39 (0.78, 2.47)	
**Change in knowledge score**	1.01 (1.00, 1.03)	1.01 (0.99, 1.02)

* Adjusted for clustering of healthcare workers in hospitals

^¶^Internal Medicine, Surgery, Pharmacy, and Administration

#### Healthcare provider characteristics

Participants who had recently graduated from their professional training were more likely to pass the practical skills assessment (aOR = 9.62; 95% CI 1.20, 76.85) than those with more than one year away from graduation (Table 5). At the completion of the ETAT+ course, on average generalist and specialist MDs demonstrated a greater improvement in knowledge (aß = 6.5; 95% CI 3.2, 9.8) and (aß = 13.5; 95% CI 8.1 18.9) respectively, higher than nurses (Table 4). Similarly, profession was associated with performance on practical skills assessment with midwives, generalist and specialist MDs having higher odds of passing than nurses (aOR = 3.12; 95% CI 0.85, 11.37), (aOR = 3.50; 95% CI 1.40, 8.73) and (aOR = 4.87; 95% CI 0.60, 39.32), respectively (Table 5). However, the relationship is statistically significant in the multivariate analysis only for generalist MD.

Compared to participants who identified as being proficient only in French, on average, those who identified as proficient in both English and French (bilingual) demonstrated a greater improvement in knowledge (aß = 7.3; 95% CI 4.3, 10.2) (Table 4). Likewise, bilingual participants had higher odds of passing the practical skills assessment (aOR = 2.60; 95% CI 1.25, 5.40) than those who identified as proficient in French (Table 5). The higher the improvement in knowledge, the higher the odds of passing the practical skills assessment (aOR = 1.01; 95% CI 0.99, 1.02) (Table 5).

Since nurses from national referral and district hospitals may have an A2 (just secondary school diploma and the lowest available qualification), A1 (three years of post-secondary nursing training) or A0 (four years of post-secondary nursing training) certification, and health facilities located in rural areas usually have the highest proportion of A2 nurses, a further stratified analysis was conducted to investigate whether performance of nurses varied by location of health facility. However, we found that nurses from urban and rural hospitals performed similarly.

#### ETAT+ course organization characteristics

Of all ETAT+ course organization related factors (Table 2), time of receipt of course materials was most strongly associated with improvement in knowledge in the adjusted model, with participants who received materials within a week of the start of ETAT+ course achieving a greater improvement (aß = 2.1; 95% CI 0.9, 5.3) than those who received materials more than one week (Table 4). Moreover, location of the ETAT+ course appeared to be strongly associated with performance on skills assessment, with participants attending an ETAT+ course outside their health facility (off-site) having higher odds of passing the practical skills assessment (aOR = 2.11; 95% CI 1.01, 4.44) than those who attended a course held within their affiliated health facility (Table 5).

Further analysis to investigate the association between performance and type of course materials stratified by hospital location found that participants who were working in rural hospitals and received printed course materials (hardcopy) had a higher performance in the pre-course knowledge assessment than those who got just e-copy (p<0.0001), but there was no difference in post course knowledge and skills assessment (P>0.05).

## Discussion

The current study demonstrates that the ETAT+ course is associated with improvements in both knowledge and skills in course attendees in Rwanda. Overall, the course participants performed better overall on practical skills assessment than on knowledge assessment. Further, the current analysis provides evidence of factors associated with knowledge and practical skills performance including the provision of pre-course materials (timing and format), location of ETAT+ course and HCWs’ language proficiency.

Consistent with previous studies, participating in continuing medical education (CME) courses including ETAT+ is associated with immediate improvement knowledge across a wide variety of professions (e.g. physicians, nurses, midwives, in training healthcare professionals) following the training [9, 15–17]. While the pass rate in the post-course knowledge assessment was high (78.0%), the overall mean post-test score was only 58.0% (SD 13.4), far below the average score of 71% for Rwandan medical students who undertook the same course and assessment [15]. Similarly, Rwandan medical students had a higher performance on the skills assessment than the HCWs (pass rate of 98% versus 78.6%) [15]. This difference might be explained by language barriers and limited access to computers and the internet among HCWs, but also eagerness/ motivation for students as they were trained in the ETAT+ during their pediatric rotation. The ETAT+ related content is also assessed in the pediatric end rotation assessment and in the medical board examination that also likely had an effect on their performance.

Local contextual factors related to the course organization (e.g. format of course materials, time of receipt of course materials, location of ETAT+ course) and HCW’s language proficiency might explain some differences in ETAT+ course performance among Rwandan HCWs. Rwanda shifted its official language from French to English in 2008 [18], and secondary and post-secondary education and CME programs are run primarily in English. In the current study, HCWs who identified as being proficient only in French performed more poorly relative to all other participants which might be explained by challenges that HCWs who were not proficient in English (e.g. unable to adequately prepare or understand course content, if it was taught mainly in English). Use of both English and French during the course implementation might therefore offer advantages to those HCWs who identified as bilingual (French & English) over those who identified as either proficient in French or English, and help explain why bilingual HCWs demonstrated the best overall performance. Relatedly, some participants would ask questions or further clarifications in Kinyarwanda, the Rwandan mother tongue while some other participants could not communicate in Kinyarwanda, especially foreign medical and nursing graduates working in Rwanda. Furthermore, it is possible that bilingual participants might have translated for English- or French-speaking participants. If so, the translating/teaching role might have supported their learning and, therefore, might explain to some point the observed greater performance among bilingual participants. Nevertheless, the effect this might have on performance of HCWs on the ETAT+ course assessment was not assessed.

Healthcare providers working in rural based health facilities and who received printed course materials performed better on the pre-course knowledge assessment than those who were offered electronic copy (usually sent by email). This difference in performance might be explained by lower penetrance of the internet and computer ownership in rural areas compared to urban areas in Rwanda [19]. However, this difference in performance disappeared in the post course assessment and further investigation of an interaction between course materials and location of health facilities was not found to be statistically significant.

Location of the ETAT+ course was an important predictor of performance in ETAT+ course as well. While courses held within health facilities were cost saving (e.g. costs associated with accommodation of participants and course venue were saved), they were associated with a poorer performance, which may be the requirement that HCWs may be required to continue to be involved in some of the work-related activities (e.g. direct patient care, night call) during course time and could have missed material when the program is held within their institution (on-site training).

Also consistent with previous research, tasks such as observing color and tone during neonatal resuscitation or checking for signs of severe malnutrition in a child with shock due to dehydration are more likely to be missed on a manikin than when examining a real child whose nutrition status, tone or color would be apparent clinically [15]. Furthermore, patients’ medical files in many health facilities in Rwanda (e.g. neonatal admission record) have checklists included, and these checklists help minimize the omission of required tasks when examining a patient. Likewise, failing to follow resuscitation sequences exactly as is required to pass the ETAT+ skills assessment may not necessarily result in resuscitation failure in practice [15].

Although nurses constitute the majority of the health workforce in Rwandan district hospitals, they unfortunately performed the most poorly on knowledge and skills assessments. This is alarming since nurses are often required to manage severely ill children and neonates without supervision. While we acknowledge that diversity in course participants (e.g. integration of multiple professions) might improve the learning experience as participants share different experiences and learn how to work together in a team, a different approach for delivering ETAT+ for nurses may improve their performance.

Better performance on the infection component compared to other ETAT+ components in the pre-course knowledge assessment might be explained by existing disease-specific programs focusing on training HCWs on management of malaria and HIV across the country, and the treatment of pneumonia as part of an integrated management of childhood illness program. We believe that integrating pediatric advanced life support courses such as ETAT+ into the ongoing Rwanda continuing professional development program might help regular training and retraining of HCWs and increase their knowledge and skills with regards to managing newborn and child health emergencies. The closer to graduation the better performance, which might suggest that the most-recently trained participants have previously (and recently) studied the ETAT+ content in their professional school training. While refresher training for HCWs in life support management cannot be overemphasized because the decay of knowledge and loss of skills pose a real threat to success of best practice care interventions [20, 21], we can speculate that better performance among HCWs who had previously attended the ETAT+ course (while still in their professional training) reflects some levels of knowledge and skills retention.

The current study was the first to assess factors associated with performance on the ETAT+ course that we are aware of, and thus provides evidence of the local contextual factors that might affect Rwandan HCWs’ performance in ETAT+ course. Despite the study sample size of 374 HCWs from 24 health facilities including three of the four national referral hospitals and 21 of Rwanda’s 40 district hospitals, our findings may not be representative of all HCWs in Rwanda given that participants and hospitals were purposefully selected–i.e. selection bias could not be ruled out. Moreover, given the current study design, single group threats to internal validity are of concerns (e.g. testing and instrumentation threats) [22]. It is possible that the improvement in knowledge observed among participants was not only due to attending the ETAT+ course. For example, taking only the pre-course knowledge assessment might affect how participants do on the post-course knowledge assessment even without participating in the ETAT+ course (testing threat) as they might memorize questions. Including different questions in the post-course knowledge assessment while still testing the same concept might have minimized the testing threat; however, changing questions might have introduced an instrumentation threat.

While program evaluation using specific metrics may be easier and more practical than trying to evaluate patient outcomes, the latter is the ultimate goal. Nevertheless, before investing the time and resources to evaluate patient outcomes, programmatic evaluation was carried out under the premise that lack of positive outcomes at the programmatic level likely means no effect on patient outcomes. Since a positive impact of the course on knowledge change and skills performance was identified among HCWs, future studies should attempt patient level evaluation to determine whether the knowledge and skills acquired translate to practice and contribute to improving patient health outcomes.

In conclusion, the current study findings shows a positive impact of the ETAT+ course on improving participants’ knowledge and skills related to managing emergency pediatric and neonatal conditions. Moreover, the analysis provides evidence of specific factors associated with improvement in both knowledge and practical skills in Rwandan HCWs. These factors are modifiable and can therefore be used to inform improvements in the delivery of the ETAT+ program in Rwanda thereby positively impacting HCWs knowledge and skills development. The findings can inform not only ETAT+ course program implementers but also other CME program implementers and policy and decision makers of key contextual factors that might affect Rwandan HCWs’ performance in CME trainings and thus take them into account when developing and implementing CME in Rwanda and regionally.
